# Epigenetic Regulation by lncRNA GAS5/miRNA/mRNA Network in Human Diseases

**DOI:** 10.3390/ijms26031377

**Published:** 2025-02-06

**Authors:** Lam Ngoc Thao Nguyen, Jaeden S. Pyburn, Nhat Lam Nguyen, Madison B. Schank, Juan Zhao, Ling Wang, Tabitha O. Leshaodo, Mohamed El Gazzar, Jonathan P. Moorman, Zhi Q. Yao

**Affiliations:** 1 Center of Excellence in Inflammation, Infectious Disease and Immunity, James H. Quillen College of Medicine, East Tennessee State University, Johnson City, TN 37614, USA; ngocthaolam.nguyen@nih.gov (L.N.T.N.); pyburn@etsu.edu (J.S.P.); lam.nguyen@nih.gov (N.L.N.); niecem@etsu.edu (M.B.S.); zhaoj2@etsu.edu (J.Z.); wangl3@etsu.edu (L.W.); leshaodo@etsu.edu (T.O.L.); elgazzar@etsu.edu (M.E.G.); moorman@etsu.edu (J.P.M.); 2 Department of Internal Medicine, Division of Infectious, Inflammatory and Immunologic Diseases, Quillen College of Medicine, East Tennessee State University, Johnson City, TN 37614, USA; 3 Hepatitis (HCV/HBV/HIV) Program, James H. Quillen VA Medical Center, Department of Veterans Affairs, Johnson City, TN 37614, USA

**Keywords:** lncRNAs, GAS5, miRNAs, epigenetic, immune regulation, human disease

## Abstract

The interplay between long noncoding RNAs (lncRNAs) and microRNAs (miRNAs) is crucial in the epigenetic regulation of mRNA and protein expression, impacting the development and progression of a plethora of human diseases, such as cancer, cardiovascular disease, inflammatory-associated diseases, and viral infection. Among the many lncRNAs, growth arrest-specific 5 (GAS5) has garnered substantial attention for its evident role in the regulation of significant biological processes such as proliferation, differentiation, senescence, and apoptosis. Through miRNA-mediated signaling pathways, GAS5 modulates disease progression in a cell-type-specific manner, typically by influencing proteins involved in inflammation and cell death. While GAS5 is recognized as a tumor suppressor in cancer, recent reports highlight its broader regulatory capacity in non-cancerous diseases. Its modulation of protein expression through the GAS5/miRNA network has been shown to both mitigate and exacerbate disease, depending on the specific context. Furthermore, the therapeutic potential of GAS5 manipulation, via knockdown or overexpression, offers promising avenues for targeted interventions across human diseases. This review explores the dualistic impacts of the GAS5/miRNA network in conditions such as cancer, cardiovascular disease, viral infections, and inflammatory disorders. Through the evaluation of current evidence, we aim to provide insight into GAS5’s biological functions and its implications for future research and therapeutic development.

## 1. Introduction

The human genome consists of coding and noncoding sequences. Coding sequences follow the central dogma “DNA makes RNA, and RNA makes protein”; however, coding sequences only account for approximately 2% of the human genome. The vast majority of the human genome is composed of noncoding sequences, which are classified based on their transcript lengths in nucleotides (nt). Transcripts of less than 200 nt are classified as small noncoding RNAs, including microRNAs (miRNAs, typically 22 nt) [1,2], small nucleolar RNAs (snorRNAs), small interfering RNAs (siRNAs), transfer-derived RNAs (tRFs), small nuclear RNAs (snoRNAs), and PIWI-interacting RNAs (piRNAs). Sequences of more than 200 nt are termed long noncoding RNAs (lncRNAs) [3,4]. It is well known that miRNAs play crucial roles in the epigenetic regulation of various cellular biological and pathophysiological pathways [5,6,7,8,9]. While significant advancements have been made in elucidating the roles of lncRNAs in human disease, their functions are highly diverse, varying from transcriptional to post-transcriptional gene regulation, and context-dependent, with their mechanisms of action, particularly lncRNA-miRNA axes, undergoing significant investigation.

Growth arrest-specific 5 (GAS5) is one of the most well-studied lncRNAs and was identified in 1988 [10]. GAS5 is a lncRNA with a transcript length of roughly 630 nt and is encoded by a DNA sequence located on chromosome 1q25 [11]. The gene comprises 12 exons, which are not well conserved because of rapid evolution in higher species. GAS5 introns encode 10 snorRNAs and 2 mature lncRNA isoforms, GAS5a and GAS5b, with their only difference being the length of exon 7 (77 nt and 45 nt, respectively) [12,13]. Given its crucial role in the regulation of cellular proliferation and differentiation, GAS5 is deemed a novel tumor suppressor in various cancers [14,15,16,17,18]. Accumulating evidence suggests that GAS5 may also play a crucial role in the pathophysiological processes occurring in non-cancer diseases, especially inflammation and infectious diseases [19,20,21,22,23].

GAS5 can influence signaling pathways through three modes of action [24]. This review will largely focus on the first mode of action, where GAS5 serves as a decoy, acting as a molecular sponge that can bind directly to its target protein or RNA to disrupt any downstream activities. For example, GAS5 acts as a decoy hormone response element for the glucocorticoid receptor, thus suppressing gene expression [12,25]. Also, GAS5 acts as a biological sponge for miRNAs, suppressing their epigenetic activities and thus directly regulating pathways mediated by miRNAs [26,27]. Moreover, GAS5 acts directly as a signaling molecule, being transcribed in response to specific triggers to facilitate downstream signal transmission. This role has been demonstrated in human colorectal cancer, where, following DNA damage, GAS5 upregulates snoRNAs to subsequently activate the p53 signaling pathway, mitigating DNA damage [24,28]. Furthermore, GAS5 has the ability to function as a transport molecule. This action requires GAS5 to bind to its target protein, which it then guides to a distinct DNA sequence where the protein can regulate transcription. Specifically, GAS5 has been demonstrated to enhance the binding of E2F transcription factor 1 (E2F1) to the promoter of the cyclin-dependent kinase inhibitor 1B (P27kip1), thereby activating P27^kip1^ [29].

In this review, we summarize the role of GAS5/miRNA axes in cancers, cardiovascular diseases, inflammatory diseases, and viral infections. This is followed by a brief discussion of the impact of GAS5, independent of miRNA-mediated axes, on these conditions. The objective of this article is to provide prospective insight for future studies on the biology of GAS5 and its pathophysiological functions in human diseases.

## 2. Role of GAS5/miRNA Axes in Cancers

GAS5 is known as a regulator of cellular differentiation and proliferation, thus targeting GAS5 and its regulated pathways has been considered a novel therapeutic treatment in cancer [16,30].

To date, the GAS5/miR-21 axis is one of the most extensively studied lncRNA-miRNA-regulated signaling pathways and has been shown to play a crucial role in the progression of various cancers via downregulated GAS5 and upregulated miR-21 expressions. GAS5 and miR-21 reciprocally regulate each other’s expression, exhibiting oncogenic roles in different human cancer diseases [31]. Specifically, the overexpression of GAS5 decreases the level of miR-21, whereas the overexpression of miR-21 eliminates GAS5-mediated regulatory effects [13,32]. Mechanistically, miR-21 targets a binding site in exon 4 of GAS5, forming a feedback loop that controls their expression via the RNA-induced silencing complex (RISC) [13]. This interaction exemplifies the miRNA-mediated silencing of target RNAs [13]. Thus, the reciprocal regulation between GAS5 and miR-21 emphasizes their critical roles in modulating cellular differentiation and proliferation, which are pivotal processes in cancer progression.

Recently, miR-21 upregulation has been identified as a powerful biomarker in various cancers, since miR-21 is known as a cell death and differentiation modulator, both enhancing cell activation/growth and suppressing cell apoptosis regulatory pathways. For instance, miR-21 has been demonstrated to inhibit the expression of one of the most potent tumor suppressor proteins—Phosphatase and Tensin Homolog (PTEN) [33]. PTEN plays a critical role in tumor suppression through phosphatase-dependent and -independent activities, with one of its more prominent roles being using its phosphatase activity to regulate a crucial pro-survival and cell growth pathway in cancers, i.e., Phosphoinositide 3-Kinase/Phosphorylated-protein Kinase B/Mammalian Target of Rapamycin (PI3KAKT/mTOR) [34]. The miR-21-mediated inhibition of PTEN in the PTEN/Phosphoinositide 3-Kinases (PI3K) pathway results in enhanced cellular growth and differentiation. The repression of PTEN derepresses Phosphorylated-protein Kinase B, also known as AKT, leading to an enhanced expression of growth-promoting protein Mammalian Target of Rapamycin (mTOR) [35]. mTOR signaling, which governs critical processes like cell cycle, proliferation, growth, survival, protein synthesis, and glucose metabolism, is frequently dysregulated in cancers, with studies showing its enhancement in various malignancies and involvement in nearly 30% of solid tumors, making it one of the most commonly affected pathways [36]. MiR-21 also regulates cell growth via regulating the Sprouty RTK signaling antagonist 1 (SPRY1)/rapidly accelerated fibrosarcoma (RAF)/extracellular signal-regulated kinase (ERK) pathway. The repression of SPRY1/2 by miR21 leads to the upregulation of RAF and ERK proteins, thus promoting cellular proliferation and differentiation in many mammalian cells [37]. Additionally, the miR-21-mediated suppression of apoptosis has been linked to the suppression of programmed cell death 4 (PDCD4), resulting in continuous cell cycle progression [38,39]. MiR-21 also directly targets and inhibits SMAD family member 7 (SMAD7), a pro-apoptosis protein, resulting in reduced apoptosis and cell death [40]. Given its role in cell growth and proliferation, miR-21 upregulation serves as a biomarker in cancer diseases.

Due to its inverse correlation with miR-21, GAS5 repression has been characterized as a biomarker in various cancers [41]. A recent study highlighted a significant repression of GAS5 in osteosarcoma patients, as well as a more prominent repression in patients with lung metastatic cancer. Upon further characterization in osteosarcoma cell lines, the GAS5 repression corresponded to upregulated epithelial–mesenchymal transition (EMT) [42], a phenomenon which increases migration and invasion, both integral for metastasis. Interestingly, others have demonstrated a suppression of the EMT process within uveal melanoma (UM) cells that was observed upon the upregulation of GAS5, reducing the overall risk of metastasis [43]. Experiments within xenograft mice models have confirmed the crucial role of GAS5 as a tumor suppressor due to its ability to act as a competing endogenous (ceRNA) for miR-21, sponging the potent pro-cancer regulator. The overexpression of GAS5 resulted in a reduced survival rate of breast cancer cell lines, as well as a decrease in the size of tumors in mice, whereas the inhibition of GAS5 displayed an opposite effect [13]. GAS5 overexpression has also been demonstrated to repress endometrial cancer formation in immunodeficient mice models through PTEN/AKT signaling and directly repressing oncogenic yes-associated protein 1 (YAP1) in tumor-associated macrophages, transforming them into an anti-tumor phenotype [44]. Additional findings support a tumor-promoting function of the aberrant GAS5/miR-21 axis via the PTEN/PIK3 or SPRY1/p21 pathways in hepatocellular carcinoma, oral squamous cell carcinoma, bladder cancer cells, and ovarian cancer [45,46,47,48,49]. Based on these studies, it is believed that the suppression of miR-21 and upregulation of GAS5 have therapeutic potential for the treatment of cancer diseases.

However, GAS5’s tumor-suppressing function extends beyond its inverse correlation with miR-21, as it also modulates cancer progression through various miRNA-mediated signaling pathways. For instance, GAS5 modulates the malignant phenotype, migration, and invasion of glioma cells via binding and suppressing miR-18-a-5p [50]. Deletion analysis detected binding sites for miR-18a-5p on the second exon of GAS5, further confirming their interaction. Another study showed that the natural compound α-Solanine exhibits anti-cancer activity and enhances radio-sensitivity in prostate cancer. Interestingly, enhanced apoptosis and DNA damage were observed, along with an increase in GAS5 level and decreased miR-18a-5p expression, in prostate cancer cells treated with α-Solanine, suggesting that targeting GAS5/miR-18a-5p can be utilized to treat prostate cancer in the presence of α-Solanine [51]. In addition, another study suggested that GAS5, miR-196a-5p, and Forkhead Box Protein O1 (FOXO1) form a positive feedback loop in glioma stem cells [52]. GAS5 increases the level of FOXO1 by inhibiting miR-196a, thus suppressing cancer invasion. Moreover, the GAS5/miR-196 axis increases the level of phosphotyrosine interaction domain containing 1 (PID1) protein, thus inhibiting glioma stem cell tumorigenicity and growth. Interestingly, FOXO1 promotes the transcription of GAS5 as well, thereby forming a positive feedback loop that can be used as an approach to treat glioma cancer. Most recently, the upregulation of GAS5 has been demonstrated to repress miR-135b-5p, resulting in the upregulation of the adenomatous polyposis coli (APC) gene, a known tumor suppressor, through its regulation of the Wnt/β-catenin pathway [53,54]. Furthermore, the tumor-suppressing function of GAS5 was reported in esophageal squamous cell carcinoma [53], in cervical cancer through the miR-196a/miR-205/FOXO1 axis [54], and in ovarian cancer via the miR-196a/Homeobox 5 (HOXA5) axis [55].

The role of GAS5 and its associated miRNAs in the regulation of cellular proliferation and differentiation has been further demonstrated by GAS5 reducing the migration and invasion of pancreatic cancer via miR-221 repression, specifically through increasing the level of suppressor of cytokine signaling 3 (SOCS3) protein [56]. The GAS5-mediated inhibition of miR-221 also results in an increased expression of the aplasia ras homolog member I (ARHI), thereby preventing the EMT process in osteosarcoma cells [57]. The inhibition of miR-221 via GAS5 sponging has also been demonstrated to upregulate tumor protein p63 (TP63), a central tumor suppressor in non-small cell lung cancer (NSCLC) [58]. Additionally, the sponging of miR-221 via GAS5 has been demonstrated to mitigate Adriamycin resistance in breast cancer, specifically through the upregulation of Dickkopf 2 (DDK2) and suppression of P-glycoprotein (ABCB1) expression and the Wnt/β-catenin pathway [59]. The GAS5/miR-222 axis has been suggested to inhibit the BCL-2-modifying factor (BMF) and its downstream BAX protein, thereby delaying the proliferation of glioma cells [60]. Notably, the proliferation of osteosarcoma cells decreased following the activation of the GAS5/miR-23a axis via the PTEN/PI3K/AKT pathway [61]. The GAS5/miR-222 axis also regulates the PTEN/AKT/mTOR pathway to suppress the progression of other cancers, such as papillary thyroid carcinoma [62], human B lymphocytic leukemia [63], and gastric cancer [64]. Additionally, recent evidence suggests that a modulation of GAS5 can alter the proliferation rates of cancer cells, specifically through the use of GAS5-enriched extracellular vesicles (EVs) as a delivery system. A recent study demonstrated the ability of sorafenib-induced EVs, which were enriched with tumor suppressor ncRNAs such as GAS5, to significantly reduce xenograft tumor area, suppress angiogenesis, and reduce the number of micrometastases in the tails of zebra fish [65].

Not only is GAS5 an effective tumor suppressor through its ability to regulate proliferation and differentiation, but GAS5 has also been demonstrated to inhibit cancer progression by driving cancer cells towards programmed cell death through autophagy. Recent findings in 293T cells emphasize the pivotal role of the GAS5/miR-23a/ATG axis in the modulation of autophagy. GAS5 suppresses the expression of miR-23a, subsequently elevating the levels of key autophagy-related proteins such as LC3II, Beclin1, and the autophagy-related 5 and 12 complex formation (ATG5-ATG12). This cascade facilitates enhanced autophagic activity. Concurrently, GAS5 overexpression leads to the repression of mTOR and p62, critical regulators of cell survival and ubiquitin-binding protein dynamics, respectively [66]. The GAS5/miR-23a axis also acts as a tumor suppressor in non-small cell lung cancer [67], ovarian cancer [68], and glioma [69].

In addition to the miRNAs mentioned, GAS5 also interacts with several other miRNAs involved in the regulation of cellular growth and apoptosis in many other contexts. For instance, by inhibiting the expression of miR-103 and miR-32-5p, GAS5 induces the PTEN/PI3K/AKT pathway, thereby suppressing the tumor progression of endometrial cancer [70] and pancreatic cancer cells [71]. GAS5 also acts as a tumor suppressor in various cancers through functioning as a ceRNA for many miRNAs, which can exacerbate conditions by contributing to the acceleration of cancer. The GAS5-mediated sponging of miRNAs includes targets such as miR-223 in renal cell carcinoma [72]; miR-135b [73] or miR-1323 [74] in hepatocellular carcinoma; miR-106b in cervical cancer [75]; miR-182-5p in colorectal cancer [76]; miR-181c-5p in pancreatic cancer [77]; miR-424 in multiple malignant phenotypes of glioma [78]; miR-145 in prostate cancer [79]; miR-135b in non-small cell lung cancer [80]; and miR-34a in solid tumors [81]. These interactions underscore the role of GAS5 as a versatile tumor suppressor across multiple cancer models. By acting as a ceRNA, GAS5 effectively derepresses either the expression of tumor suppressor genes or critical regulatory pathways that inhibit tumor proliferation, invasion, and resistance to therapy. This highlights the broad therapeutic potential of targeting the GAS5/miRNA axis to modulate cancer progression and improve treatment outcomes.

**Figure 1:**
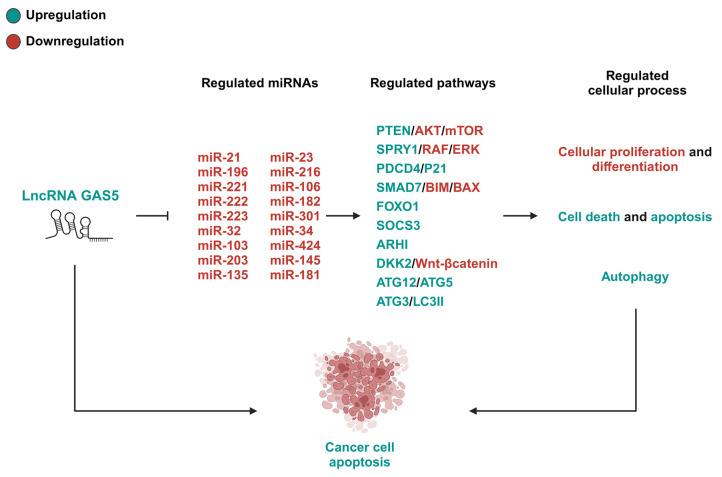
The GAS5/miRNA-regulated pathways in cancers. This figure depicts the pathways modulated by the GAS5/miRNA-mediated mechanisms that regulate the progression of cancers. The upregulation of GAS5 results in the downregulation of its target miRNAs, thereby regulating downstream proteins involved in cellular activation, proliferation, differentiation or apoptosis, and cell cycle arrest. Thus, increasing GAS5 levels is considered a promising therapeutic approach for suppressing the progression of various cancers. PTEN, phosphatase and tensin homolog; AKT, protein kinase B; mTOR, mammalian target of rapamycin; SPRY1, Sprouty RTK signaling antagonist 1; RAF, rapidly accelerated fibrosarcoma; ERK, extracellular signal-regulated kinase; PDCD4, programmed cell death 4; P21, cyclin-dependent kinase inhibitor 1; SMAD7, SMAD family member 7; BIM, Bcl-2-like protein 11; BAX, BCL2-associated X; FOXO1, forkhead box protein O1; SOCS3, suppressor of cytokine signaling 3; ARHI, aplasia Ras homolog member I; DKK2, Dickkopf 2; ATG12, autophagy related 12; ATG3, autophagy related 3; ATG8, autophagy related 8; LC3II, LC3-phosphatidylethanolamine conjugate.

GAS5/miRNA axes play significant roles in modulating cellular proliferation, differentiation, and apoptosis, allowing them to have a pivotal tumor-suppressive role in cancer progression. The GAS5/miR-21 axis is particularly well-characterized and highlighted by the reciprocal regulation between GAS5 and miR-21 and their impact on prominent oncogenic pathways, such as PTEN/PI3K/AKT or SPRY/RAF/ERK [13,31,32,35,37,38,39,40]. GAS5’s tumor-suppressive ability is not limited to this axis, as it functions as a ceRNA for many other miRNAs such as miR-18a-5p, miR-196a, miR-221, and miR-23a, among others, to inhibit hallmark cancer characteristics such as EMT [42,43,56,57], chemoresistance [46,59,77], and metastasis [63,71] across a multitude of cancer cell types. Altogether, these findings emphasize GAS5’s multifaceted role as a ceRNA, sponging various oncogenic miRNAs, and display the therapeutic potential of targeting GAS5/miRNA axes, through the upregulation of GAS5, to suppress tumorigenesis and metastasis across numerous forms of cancer (see Figure 1 and Table 1).

## 3. GAS5/miRNA Axes and Cardiovascular Diseases

As previously discussed, the GAS5/miR-21 axis has been well defined in cancer studies, and unsurprisingly it has also been a significant area of focus in other areas of biomedical research, such as cardiovascular disease. The aberrant expression of GAS5 and miR-21 is associated with cardiovascular disease and dysfunctions such as cardiac fibrosis [87], acute myocardial infarction [85], increased cardiomyocyte damage and apoptosis [86,89], and the aberrant proliferation and migration of vascular smooth muscle cells [90].

Beyond miR-21, GAS5 has been shown to exacerbate various cardiovascular diseases by modulating other miRNAs and their downstream signaling pathways, with studies highlighting the therapeutic potential of GAS5 knockdown or knockout strategies. One study regarding myocardial infarction suggested that by sponging miR-525-5p, GAS5 enhances the expression of calmodulin 2 (CALM2) and induces the apoptosis of myocardial cells, thereby promoting the development and progression of myocardial infarction [121]. In another study, GAS5 acted as a biological sponge for miR-142-5p in cardiomyocyte H9c2 cells, thereby suppressing miR-142-5p activity by targeting the tumor protein P53 inducible nuclear protein 1 (TP53INP1), which resulted in an increase in cell proliferation via the PI3K/AKT and MEK/ERK signaling pathways. Additionally, GAS5 silencing reduces the levels of p53, Bax, and cleaved caspase-3 proteins, while also increasing the expression of cyclinD1, cyclin-dependent Kinase 4 (CDK4), and B-cell lymphoma 2 (BCL-2) proteins, collectively leading to a decrease in cell apoptosis. Thus, GAS5 silencing protects cardiomyocytes against hypoxic injury via sponging miR-142-5p [105]. Importantly, GAS5 also modulates hypoxia/reoxygenation (H/R)-induced cardiomyocytes. GAS5 acts as a ceRNA for miR-335, as knocking down GAS5 leads to the repression of the miR-335 target protein Rho-associated protein kinase 1 (ROCK1). In turn, the repression of ROCK1 activates the PI3K/AKT pathway and inhibits glycogen synthase kinase 3 (GSK-3β) and mitochondrial permeability transition pore (mPTP) from opening proteins. Altogether, these changes shift the balance toward pro-survival pathways and mitochondrial stability, leading to the silencing of GAS5, which limits myocardial infarct size and reduces apoptosis in H/R-induced cardiomyocytes [117]. By targeting miR-135a, GAS5 can impact the progression of atherosclerosis (AS). In a recent study, THP-1 macrophages treated with oxidized low-density lipoprotein (ox-LDL) and apolipoprotein E (apoE)−/− mice on a high-fat diet (HFD) were used as vitro and in vivo models, respectively, to investigate the role of GAS5 in atherosclerosis. The results showed that GAS5 levels increased in LDL-treated macrophages as well as in AS mice. Notably, GAS5 silencing induced miR-135a, decreasing inflammation and AS disease progression [103]. This role of GAS5 in atherosclerosis progression is further supported by a study investigating the role of GAS5 in coronary AS through the miR-194-3p/Thioredoxin-interacting protein (TXNIP) axis. This study demonstrated that elevated GAS5 and TXNIP expression, as well as reduced miR-194-3p expression, corresponded with increased endothelial cell (EC) apoptosis. Upon the inhibition of GAS5 or overexpression of miR-194-3p, significant enhancement of endothelial cell proliferation and a reduction in apoptosis via TXNIP were observed, providing promising potential for reducing the formation of atherosclerotic plaques in AS [110].

While there is an accumulation of evidence suggesting an overall negative role for GAS5 in cardiovascular health, recent reports also suggest a protective role in some contexts. Notably, GAS5 was identified as an anti-senescence lncRNA in endothelial progenitor cells (EPCs). It was reported to function as a ceRNA of miR-223. Upon knocking down GAS5, miR-223 disrupts proliferation and enhances senescence in EPCs through the suppression of the nicotinamide phosphoribosyltransferase (NAMPT) and PI3K/AKT pathways [114]. Recent reports have demonstrated certain protective effects of GAS5 against cardiac fibrosis through the regulation of the miR-217/Sirtuin 1 (SIRT1) pathway. One study utilized isoprenaline-induced myocardial fibrosis in mice to demonstrate that GAS5 overexpression significantly reduced the expression levels of collagen, NOD-like receptor family pyrin domain containing 3 (NLRP3), Caspase-1, and IL-1β, leading to an overall reduction in inflammatory response and pyroptosis. Conversely, the protective effect of GAS5 was ablated following the overexpression of miR-217, which downregulates SIRT1 and promotes NLRP3 inflammasome activation. Altogether, these findings suggest a critical role for the GAS5/miR-217/SIRT1 axis in restraining cardiac fibrosis, leading to the preservation of cardiac function [112]. Furthermore, GAS5 has been shown to play a protective role in myocardial injury in rats with adjuvant arthritis by downregulating the miR-21/ Toll-like-Receptor 4 (TLR4)/NLRP3 axis, resulting in reduced inflammation, pyroptosis, and myocardial damage [91]. These findings underscore GAS5’s broader potential as a regulator of inflammatory and fibrotic processes within the cardiovascular system.

**Figure 2:**
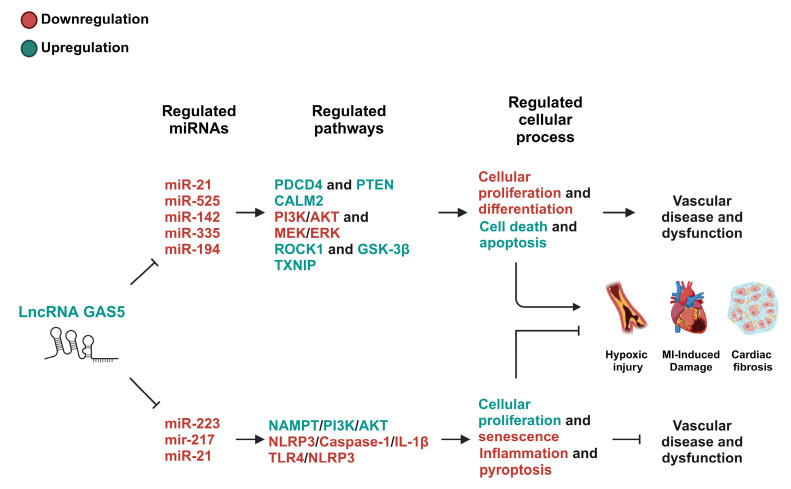
The GAS5/miRNA-regulated pathways in cardiovascular diseases. This figure depicts the pathways modulated by GAS5/miRNA-mediated mechanisms that have either debilitating or protective effects on cardiovascular diseases. The pathways involved in modulating cell activity in a debilitating manner exert their effects, in general, through the suppression of cellular proliferation and differentiation while promoting cell death and apoptosis. The pathways involved in modulating cell activity in a protective manner exert their effects, in general, through the suppression of senescence, inflammation, or pyroptosis while promoting cellular proliferation. These pathways are largely cell-type- and context-dependent. PDCD4, programmed cell death protein 4; PTEN, phosphatase and tensin homolog; PI3K, phosphoinositide 3-kinase; AKT, protein kinase B; MEK, mitogen-activated protein kinase; ERK, extracellular signal-regulated kinase; ROCK1, rho-associated coiled-coil containing protein kinase 1; GSK-3β, glycogen synthase kinase 3 beta; TXNIP, thioredoxin-interacting protein; NAMPT, nicotinamide phosphoribosyltransferase; NLRP3, NOD-, LRR-, and pyrin domain-containing protein 3; TLR4, toll-like receptor 4; MI, myocardial infarction.

The role of GAS5 in cardiovascular disease highlights its complex and context-dependent interplay with miRNA and downstream pathways. While GAS5 has been demonstrated to function in a manner that exacerbates conditions like myocardial infarction [121], atherosclerosis [103], and hypoxia/reoxygenation injury [117] by promoting apoptosis, inflammation, and fibrosis, it also exhibits protective effects, such as mitigating cardiac fibrosis [112] and countering endothelial progenitor cell senescence [114]. This duality stresses the need for tailored therapeutic approaches targeting GAS5. Leveraging its role as a ceRNA offers significant potential for precision RNA-based therapies to modulate miRNA activity in an effective manner. Future research should aim to clarify GAS5’s mechanisms across a myriad of cardiovascular disease models to advance novel treatments within this field (see Figure 2 and Table 1).

## 4. Roles of GAS5/miRNA Axes in Inflammation-Associated Diseases

In addition to its roles in cancers and vascular diseases, GAS5 also plays a crucial role in regulating the progression of many inflammation-driven diseases.

GAS5 appears to have a distinctly detrimental role in the context of multiple inflammation-related neurological diseases. A previous study has shown that the inhibition of miR-221 by GAS5 induces the transcription of p53-upregulated modulator of apoptosis (PUMA) and its downstream proteins such as c-Jun N-terminal kinase (JNK)-H2A histone family member X (H2AX), thereby promoting neuronal apoptosis under hypoxic conditions [27]. Another study demonstrated that GAS5 levels are upregulated in mice with cerebral ischemic stroke (CIS), a leading cause of neurological disability worldwide. The knockdown of GAS5 was effective at attenuating the cerebral infarct, neurological injury, apoptosis, and inflammatory response within the middle cerebral artery occlusion (MCAO) model. Specifically, GAS5 was found to directly bind to miR-9, which, when overexpressed, displayed similar protective effects to those seen in the GAS5 knockdown. Furthermore, FOXO3 was determined to be directly repressed by miR-9, and when restored, FOXO3 was shown to reverse the protective effects that miR-9 previously demonstrated. Altogether, these observations suggest GAS5 promotes ischemic stroke injury through sponging miR-9 to derepress FOXO3 [82]. Interestingly, others have examined a similar debilitating role of GAS5 in CIS, where the knockdown of GAS5 reduces the progression of CIS by regulating the miR-26b-5p/SMAD1 axis [99]. Others demonstrated that in mice undergoing MCAO surgery and oxygen–glucose deprivation (OGD) treatment, GAS5 negatively correlated with miR-137 levels. Silencing GAS5 led to an upregulation of miR-137 levels, along with a decrease in its endogenous target notch homolog 1 (Notch-1) protein. This miR-137 upregulation increased cell viability and decreased caspase-3 activation and cell apoptosis in neurons under OGD conditions. These findings indicate that GAS5 promotes the progression of CIS by acting as a ceRNA for regulating the miR-137-mediated notch-1 signaling pathway, which may provide a novel therapeutic approach for CIS [104]. Moreover, by regulating miR-21/PTEN/PI3K/AKT signaling, GAS5 promotes neural apoptosis, thereby contributing to the progression of CIS [88].

A link between GAS5 and Parkinson’s disease (PD) has been investigated using an in vitro cell model with lipopolysaccharide-induced microglia and an in vivo model established in C57BL/6 mice [115]. GAS5 has been shown to accelerate Parkinson’s disease (PD) progression by promoting inflammation in microglia through the miR-223-3p/NLRP3 axis. Specifically, GAS5 acts as a competitive sponge for miR-223-3p, thereby increasing NLRP3 expression and activating the NLRP3 inflammasome, which triggers the secretion of pro-inflammatory cytokines like interleukin-1β (IL-1β) and IL-18, further exacerbating the inflammatory response and PD progression in both in vitro and in vivo models [115]. Another study displayed an alternate pathway through which GAS5 promotes PD progression. Here, GAS5 binds and sequesters miR-150, which normally targets and represses FOSs like antigen 1 (Fosl1), thereby activating the PTEN/AKT/mTOR pathway, promoting apoptosis and inhibiting overall neuronal activity [108]. Together, these studies highlight the detrimental role of GAS5 in PD progression, which it enacts primarily through its regulation of inflammatory and apoptotic pathways.

In addition to its detrimental effects in CIS and PD, GAS5 has also been implicated to be involved in the development of other inflammation-related neurological disorders. Most recently, in Alzheimer’s disease (AD) studies, GAS5 has been found to be significantly upregulated in disease models and patient samples, where it exacerbates pathogenesis by acting as a ceRNA, sponging miR-23b-3p. This repression allows for the increased expression of GSK-3β and PTEN, critical mediators of tau hyperphosphorylation, amyloid-beta (Aβ) accumulation, and neuronal apoptosis. The GAS5/miR-23b-3p axis activates a PTEN/PI3K/Akt/GSK-3β signaling pathway, which amplifies AD-associated neurodegeneration and cognitive decline [98]. In the context of epilepsy, GAS5 interacts with miR-135a-5p to regulate KCNQ3, a potassium channel subunit vital for neuronal excitability. The upregulation of GAS5 reduces miR-135a-5p levels, decreasing KCNQ3 expression, which may increase a person’s susceptibility to seizures by destabilizing their membrane potential regulation. The knockdown of GAS5 in epileptic models mitigates seizure frequency and prolongs latency [102]. Additionally, GAS5 has been demonstrated to exacerbate the progress of neonatal hydrocephalus through its ceRNA activity on miR-325-3p, which regulates the expression of chaperonin containing T-complex protein 1, subunit 8 (CCT8), a complex that has been demonstrated to play a crucial role in protein quality control as it relates to neurological disease [116]. Consistently, the knockdown of GAS5 in the context of the inflammation-related neurological diseases appears to have substantial therapeutic potential.

GAS5 has been demonstrated to exacerbate and promote dysfunction in the context of a few other inflammation-associated diseases. For example, GAS5 is reported to induce the progression of childhood pneumonia. Elevated levels of GAS5 promote macrophage differentiation toward the M1 phenotype, which secretes a high level of pro-inflammatory cytokines, instead of the M2 phenotype, which produces anti-inflammatory cytokines such as IL-10 and transforming growth factor β (TGF-β). GAS5 promotes macrophage polarization to the M1 phenotype by regulating the miR-455-5p/SOCS3 axis. By acting as a sponge for miR-455-5p, GAS5 enhances SOCS3 expression, thereby suppressing the Janus kinase 2-signal transducer (JAK2) and activator of transcription 3 (STAT3) pathway, promoting macrophage polarization toward the M1 phenotype and thus leading to more severe cases of pneumonia in children [120]. GAS5 also promotes asthma progression. The upregulation of GAS5 levels in asthmatic airways results in a decrease in miR-10a, which upregulates brain-derived neurotrophic factor (BDNF). As a result, the proliferation rate of the airway smooth muscle cells increases, thereby thickening the airway wall. Thus, targeting the GAS5/miR-10a/BDNF regulatory axis could significantly decrease airway hyperresponsiveness and reduce the progression of asthma [83]. Additionally, GAS5 plays a crucial role in renal ischemia/reperfusion (I/R), which contributes to acute kidney injury. By inhibiting miR-21, GAS5 upregulates the miR-21 target proteins PTEN, PDCD4, or Thrombospondin-1 (TSP-1), leading to the increased apoptosis and cell death of renal epithelial cells [93].

Although much of the literature presents GAS5 as a detrimental factor that promotes the progression of various diseases, its role in other conditions is more nuanced and context-dependent. GAS5 has recently been demonstrated, in varying models, to have a multifaceted role in its involvement in inflammatory responses and mechanisms in sepsis. Recent reports have demonstrated the occurrence of GAS5 upregulation in endothelial cells through oxidative stress-activated MiT-TFE transcription factors, a phenomenon that helps protect mitochondrial function and reduce inflammation. This protective mechanism involved the GAS5-mediated sponging of miR-23a-3p, which stabilized and enhanced MITF activity and Nrf2 to preserve mitochondrial integrity and reduce lung injury in instances of sepsis [97]. Another study implicates GAS5 as a key suppressor of inflammatory responses in sepsis through the modulation of the miR-155-5p/SIRT1/HMGB1 axis [109]. While previous studies highlight a protective role for GAS5 in mitigating inflammation and preserving cellular integrity, others demonstrate the potential of GAS5 to exacerbate inflammatory damage in a different cellular context. One study found GAS5 levels to be elevated in lipopolysaccharide (LPS)-treated THP-1 cells. That study reveals that GAS5 acts as a sponge for miR-23a-3p, increasing the expression of TLR4 and thereby promoting inflammation and apoptosis in sepsis. Specifically, upon GAS5 knockdown, TLR4 overexpression mitigates the reduction in inflammatory cytokines and subsequent cell death, an indication that GAS5 enhances sepsis-related inflammatory responses through altering the miR-23a-3p/TLR4 axis [96]. Recently, others have hypothesized that GAS5 may have a dual role in sepsis, promoting inflammation in early stages and inhibiting excessive inflammatory damage in later stages through different mechanisms [97,123]. Altogether, the context-dependent nature of GAS5 and its interaction with miRNA axes in sepsis highlights its complex role, exhibiting protective effects in some cellular environments while exacerbating inflammation and apoptosis in others, potentially influenced by timing and cell-specific factors.

GAS5 has been cited to have distinct protective effects in the context of a few other inflammation-driven diseases through miRNA-mediated pathways that suppress inflammation, fibrosis, and apoptosis. For instance, in nonalcoholic fatty liver disease (NAFLD), GAS5 acts as a competing endogenous RNA (ceRNA) for miR-28a-5p, which has been demonstrated to promote pyroptosis through MARCH7-mediated NLRP3 activation, thereby exerting a protective effect [100]. In a similar fashion, GAS5 reduces the severity of rheumatoid arthritis (RA) through sponging miR-361-5p, a process which upregulates PDK4, ultimately limiting synovial hyperplasia and cell proliferation [118]. In osteoarthritis (OA), GAS5 attenuates inflammation-driven chondrocyte apoptosis through the upregulation of SMAD4 via miR-146a sponging [107]. This protective function is further exemplified by the ability of GAS5 to reduce renal fibrosis in diabetic nephropathy (DN) through sponging miR-221, which elevates SIRT1 expression, highlighting its potential role in mitigating fibrosis and promoting cellular homeostasis in the kidney [113]. The GAS5/miR-452-5p axis has also been demonstrated to have a prominent role in reducing the extent of diabetic nephropathy-related inflammation, oxidative stress, and pyroptosis in renal tubular cells [119]. Finally, in renal fibrosis independent of diabetes, GAS5 has been demonstrated to mitigate TGF-β1-induced fibrosis by modulating miR-142-5p through the Smad3 pathway [106].

**Figure 3:**
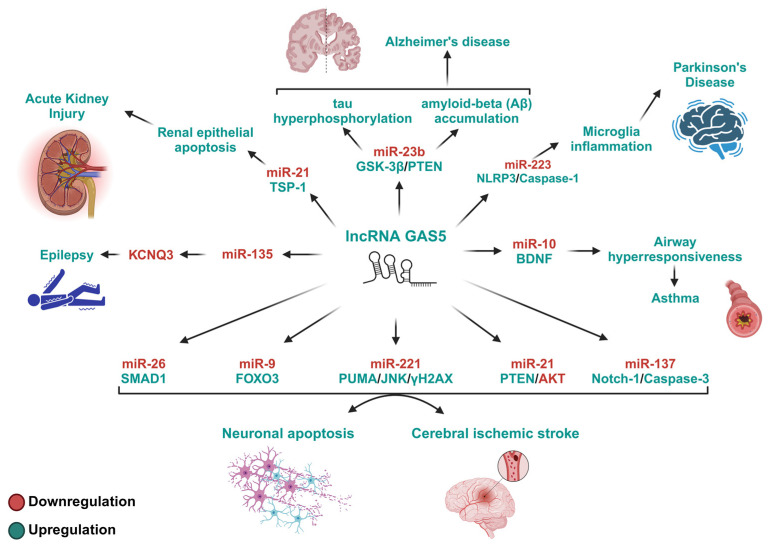
The GAS5/miRNA-regulated pathways with debilitating roles in inflammation-associated diseases. This figure depicts the pathways modulated by GAS5/miRNA-mediated mechanisms that exert debilitating effects on inflammation-associated diseases. The proteins involved in these pathways are known for their roles in cell activation, proliferation, differentiation, apoptosis, and cell cycle arrest. By inhibiting specific miRNAs and their target proteins, elevated GAS5 levels promote apoptosis and cell death, thereby promoting inflammation and apoptosis, increasing the severity of inflammatory diseases. TSP-1, thrombospondin-1; GSK-3β, glycogen synthase kinase 3; PTEN, phosphatase and tensin homolog; NLRP3, NOD-like receptor family pyrin domain containing 3; BDNF, brain-derived neurotrophic factor; SMAD1, SMAD family member 1; FOXO3, forkhead box protein O3; PUMA, p53-upregulated modulator of apoptosis; JNK, c-Jun N-terminal kinase; γH2AX, phosphorylated histone H2AX; AKT, protein kinase B; Notch-1, neurogenic locus notch homolog protein 1; KCNQ3, potassium voltage-gated channel subfamily Q member 3.

**Figure 4:**
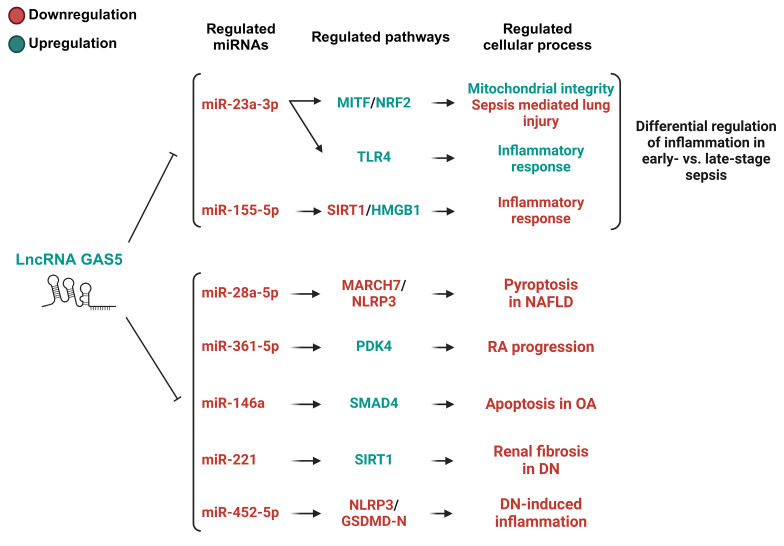
The GAS5/miRNA-regulated pathways with protective roles in inflammation-associated diseases. This figure depicts the pathways modulated by GAS5/miRNA-mediated mechanisms that exert protective effects and differential effects (sepsis) on inflammation-associated diseases. For sepsis, miRNAs appear to have a stage-dependent role in inflammation, where early sepsis relies on mitochondrial autophagy for antioxidant defense and intracellular stability, while GAS5 depletion in late-stage sepsis exacerbates inflammation, increasing tissue damage. Regarding the other disease models displayed, the overexpression of GAS5 leads to the repression of miRNAs displaying roles in inflammation, pyroptosis, proliferation, and apoptosis. This suppression of inflammation, apoptosis, and pyroptosis is significant in reducing the progression of these inflammation-associated diseases. MITF, microphthalmia-associated transcription factor; NRF2, nuclear factor erythroid 2–related factor 2; TLR4, toll-like receptor 4; SIRT1, Sirtuin 1; HMGB1, high mobility group box 1; MARCH7, membrane-associated ring-CH-type finger 7; NLRP3, NOD-like receptor family pyrin domain containing 3; PDK4, pyruvate dehydrogenase kinase 4; SMAD4, SMAD family member 4; GSDMD-N, gasdermin D N-terminal fragment; NAFLD, nonalcoholic fatty liver disease; OA, osteoarthritis; DN; diabetic nephropathy.

GAS5 plays a dual role in various diseases, displaying protective effects in some contexts and detrimental effects in others. Its promotion of inflammation and apoptosis in the context of ischemic stroke [82,88,99,104], Parkinson’s disease [108,115], and Alzheimer’s disease [98], among many other previously discussed diseases, highlights the potential of GAS5 inhibition to be used as a therapeutic strategy to mitigate inflammation, neuronal apoptosis, and disease progression. However, its protective function in diseases such as NAFLD [100], RA [118], and DN [113,119], among others, highlights the therapeutic potential of GAS5 upregulation. The case of sepsis adds complexity, where GAS5’s dual role, suppressing or encouraging inflammation in what appears to be a timing- and cell-dependent manner [96,97,109,123], offers potential opportunities for finely tuned therapeutic interventions. Overall, these findings emphasize that the therapeutic potential of GAS5 is distinctly cellular- and disease-context-dependent, suggesting that the targeted manipulation of GAS5’s expression and activity could lead to novel therapeutic strategies for a broad spectrum of inflammation-associated diseases (see Figure 3 and Figure 4 and Table 1).

## 5. GAS5/miRNA Axes in Viral Infections

While extensive research shows that GAS5 and miRNAs play important roles in viral infections, the literature exploring their interactions and the implications of these interactions in viral pathogenesis remains limited. GAS5 has been demonstrated to be downregulated in HIV infection [124]. The significance of GAS5 expression in HIV infection has been further elucidated through its role as a sponge for miR-873, which has been demonstrated to significantly alter HIV-1 replication. The upregulation of GAS5 led to a substantial repression of miR-873, resulting in decreased HIV replication. Conversely, the siRNA-mediated knockdown of GAS5 increased miR-873 levels, which correspondingly enhanced HIV infection. Additionally, the overexpression of GAS5 was found to reduce the levels of HIV-1 Gag and Pol proteins, while its knockdown resulted in elevated levels of HIV-1 mRNA. Similar results were observed for the HIV-1 p24 and Tat proteins [122]. In addition to HIV-1 proteins, GAS5 has also been shown to suppress the replication of hepatitis C virus (HCV) infection. By acting as a decoy for the viral NS3 protein, GAS5 directly suppresses HCV replication, thus decreasing the progression of HCV disease [21]. Recent reports also suggest GAS5 deficiency could be a potential biomarker for discriminating between moderate and severe SARS-CoV-2 cases. The downregulation of GAS5 allows for increased miR-200, yielding a reduction in ACE-2 which is linked to increased inflammation and cytokine storms [111]. We have recently reported that GAS5 regulates the function of CD4 T cells derived from people living with HIV (PLWH). Our data showed that increasing GAS5 in CD4 T cells restored T cell functions via repressing miR-21, which acts as an accelerator of apoptosis and senescence in CD4 T cells. Importantly, our study also suggested that in addition to the effect of miR-21-mediated signaling, other miRNAs may play a role in the regulation of CD4 T cell functions [94].

**Figure 5:**
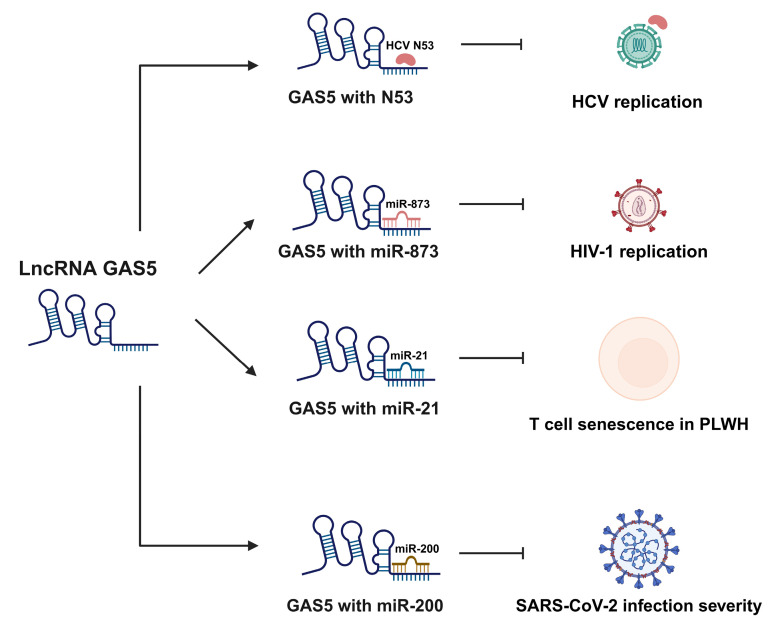
The GAS5/miRNA-mediated regulation of viral infections. This figure depicts the mechanisms by which GAS5 regulates the progression of infectious diseases caused by viral infections. GAS5 plays a critical role in regulating infectious diseases associated with HCV and HIV infections by directly binding to viral proteins or miRNAs that promote viral replication. GAS5 also demonstrates therapeutic potential in PLWH, displaying an ability to improve the activity and longevity of CD4 T cells in ART-treated PLWH. Finally, GAS5 serves as an important biomarker of severe SARS-CoV-2 infection through its ability to function as a ceRNA for miR-200. Unbound miR-200 can inhibit ACE-2 expression, a phenomenon which is linked to increased inflammation and cytokine storms in SARS-CoV-2 infection. HCV, hepatitis C virus; HIV, human immunodeficiency virus; PLWH, patients living with HIV; ART, antiretroviral therapy; ACE-2, angiotensin-converting enzyme 2.

These studies demonstrate that GAS5 plays a crucial role in regulating the immune dysregulation associated with HIV, HCV, and SARS-CoV-2. By modulating miRNA activity, GAS5 effectively suppresses viral replication, reduces inflammation, and restores immune function, specifically through functioning as a ceRNA of miR-873, miR-21, and miR-200 [21,94,111,122,124]. The ability of GAS5 to attenuate HIV- and HCV-induced immune dysfunction, as well as its potential as a biomarker of the severity of SARS-CoV-2 infections, demonstrates its potential as a target for lncRNA-based therapeutic interventions. Modulating GAS5 levels could serve as a novel strategy to mitigate disease progression and improve patient outcomes in viral infections (see Figure 5 and Table 1).

## 6. GAS5 in Human Diseases Independent of miRNA Axes

While much of this review focuses on the impact of miRNA-mediated GAS5 regulatory functions, it is crucial to understand that GAS5 itself has regulatory functions apart from serving as a ceRNA for miRNAs. Whether directly participating in the regulation of the p53 signaling pathway as a signaling molecule [28], acting as a decoy for glucocorticoid receptors (GRs) [12,25,125], or acting as a guide to promote gene transcription [29], GAS5 has been demonstrated to be involved in a myriad of biological processes at the epigenetic, transcriptional, and post-transcriptional levels [93,126,127]. This impact can be seen across a wide variety of disease presentations and is applicable in the context of cancers, cardiovascular diseases, inflammatory diseases, and viral infections, all of which have been discussed in detail throughout this review.

In general, GAS5 has been demonstrated to play significant roles in the suppression of cancers through the regulation of oncogenic signaling pathways, cell cycle progression, and cellular apoptosis [128]. Regarding oncogenic signaling pathways, GAS5 serves a critical regulator of either the PI3K/AKT/mTOR pathway or the PTEN/AKT pathway through sponging miRNAs such as miR-32 [71], miR-23a [61,95,98], miR-21 [44], and miR-222 [62,63,64]. However, the discussion of the GAS5-mediated regulation of important cell cycle mediators is not limited to the ability of GAS5 to sponge miRNAs. Indeed, GAS5 has been demonstrated to suppress c-Myc, which is required for the activation of CDKs during cell cycle progression, through cooperation with eIF4E during the translation initiation phase. This interaction prevents c-Myc mRNA from entering the polysome, subsequently blocking the translation of c-Myc. This repression of c-Myc can potentially induce cell growth arrest through reduced cyclin E/CDK2 function and the subsequent upregulation of p27 levels, a potent inducer of growth arrest [129,130]. GAS5 has also been demonstrated to have a regulatory role, seemingly independent of miRNA activity, on crucial negative regulators of cell cycle progression such as p21. One study focusing on stomach cancer identified a mechanism wherein the depletion of GAS5 increased the turnover of transcriptional activator Y-box-binding protein 1 (YBX1) through direct interaction, thereby reducing p21 expression and subsequent G1 phase arrest [131]. This novel mechanism of GAS5 suppressing stomach carcinogenesis via the YBX1/p21 pathway serves as a potential target for the development of lncRNA-based therapeutics for the treatment of stomach cancer. Additionally, GAS5 suppresses the oncogenic activity of enhancer of zeste homolog 2 (EZH2), an epigenetic modifier overexpressed in cancers like melanoma [132] and bladder cancer [18], by recruiting the transcriptional repressor E2F4 to the EZH2 promoter, thereby downregulating EZH2 expression and restoring tumor suppressor genes such as cyclin dependent kinase inhibitor 1C (CDKN1C) [133]. Finally, GAS5 displays significant regulatory functions on cellular apoptosis, specifically through its ability to function as a decoy hormone response element for the glucocorticoid receptor [12,25]. GAS5 acts as a riborepressor of the glucocorticoid receptor (GR), suppressing GR-induced transcriptional activity through its double-stranded glucocorticoid response element (GRE) mimic sequence, encoded in exon 12, which binds to the DNA binding domain of GR [12]. Through this mechanism, GAS5 overexpression has been shown to promote apoptosis in breast cancer cells [134] and increase basal and drug-induced apoptosis in prostate cancer cells, while GAS5 downregulation attenuates apoptosis [135]. Interestingly, one current clinical trial is aimed at further identifying and validating the role of GAS5 in prostate cancer through the detection and characterization of GAS5 gene polymorphisms (clinicaltrials.gov; accessed on 29 January 2025 ID: NCT06505356).

Several research groups have demonstrated the ability of GAS5 to significantly regulate cardiovascular function through mechanisms independent of miRNA axes, with GAS5 binding directly to the target protein in this case. In the context of myocardial infarction, GAS5 was demonstrated to ameliorate cardiomyocyte apoptosis and reduce infarct size in vivo by negatively regulating semaphorin 3a (sema3a) [136]. Sema3a plays a critical role in cardiac remodeling and vascular biology, as it potently inhibits angiogenesis and promotes apoptosis [137,138], both of which can exacerbate cardiac damage following infarction. GAS5 was demonstrated to directly bind to and interact with sema3a in RNA immunoprecipitation (RIP) and RNA pull-down assays [136]. Additionally, the RNA pull-down assay demonstrated GAS5’s direct interaction with a target protein. In primary varicose great saphenous veins (GSVs), GAS5 can directly bind to Annexin A2 to promote the pathogenesis of varicosities through the regulation of smooth muscle cell (SMC) proliferation, migration, and apoptosis [139]. This interaction suppresses the pro-proliferative and pro-migratory effects of Annexin A2, thereby inhibiting the excessive SMC activity that drives vascular remodeling, ultimately supporting vascular homeostasis. Additionally, GAS5 has been demonstrated to be a potent suppressor of TGF-β/Smad3 signaling in SMC differentiation, binding to Smad3 via RNA Smad-binding elements (rSBEs) and thereby preventing Smad3 from associating with DNA and inhibiting the transcription of TGF-β-responsive genes [140]. This example is noteworthy, because it demonstrates the ability of GAS5 to function as a molecular brake in terms of Smad3 activity in the basal state to maintain cellular homeostasis and refine Smad3 signaling during TGF-β induction, further highlighting the role of GAS5 as a critical regulator of SMC differentiation in various physiological contexts.

GAS5 has long been recognized for its ability to regulate inflammatory pathways through its interaction with glucocorticoid receptors (GRs) [12], making its activity independent of miRNA-mediated axes in the context of inflammation. GRs are critical in treating inflammatory conditions as they mediate the effects of glucocorticoid drugs by directly suppressing the activity of NF-κB [141], a central transcription factor in the immune and inflammatory responses. Importantly, GAS5 directly interacts with both GR and the NF-κB subunit p65, as verified by RIP assays [142]. This interaction inhibits NF-κB activity by modulating its DNA binding capacity and reducing the expression of pro-inflammatory target genes, such as TNF-α. Notably, GAS5 is dysregulated in prominent inflammatory disorders, such as rheumatoid arthritis, systemic lupus erythematosus, multiple sclerosis, and sarcoidosis [22]. Currently, there is a clinical trial aimed at investigating GAS5 expression elevation in peripheral blood mononuclear cells (PBMCs), which has been associated with steroid resistance in idiopathic nephrotic syndrome (INS), particularly in steroid-resistant nephrotic syndrome (SRNS), where GAS5 may impede GR activity and contribute to glucocorticoid insensitivity (clinicaltrials.gov; ID: NCT06325137). Given that glucocorticoids remain the pre-eminent therapeutics for the treatment of inflammatory diseases [143], the ability of GAS5 to modulate glucocorticoid sensitivity has significant therapeutic implications. The increasing prevalence of glucocorticoid resistance has significant clinical relevancy [144], and GAS5’s established role as a competitive inhibitor of GR-DNA binding suggests it may be critical in the development of steroid insensitivity. Understanding this mechanism could be particularly valuable as cases of glucocorticoid resistance continue to rise, potentially offering new therapeutic strategies to restore steroid sensitivity in resistant patients.

Finally, while the role of GAS5 in a variety of different viral infections is widely recognized, most of the mechanisms described rely on GAS5/miRNA-mediated axes [94,111,122]. However, in the context of HCV infection, this review has discussed how GAS5 can act as a decoy for the viral NS3 protein through direct RNA-to-protein interactions [21]. Similarly, in HBV-related hepatocellular carcinoma, GAS5 has been shown to function through direct RNA–protein interactions with YBX1, where its downregulation via HBx-induced methylation promotes cancer progression, demonstrating another example of GAS5’s miRNA-independent regulatory mechanisms in viral pathogenesis [145].

The above studies are indicative of the ability of GAS5 to exert a broad range of regulatory functions beyond its role as a ceRNA for miRNAs, influencing cellular processes at epigenetic, transcriptional, and post-transcriptional levels. In cancer, GAS5 functions through mechanisms such as c-Myc translation repression [129,130], YBX1/p21 pathway regulation [131], and EZH2 suppression [133], providing cellular protection in each context. In cardiovascular disease, GAS5 exhibits three key protein interactions: binding to sema3a to reduce cardiac damage and apoptosis following myocardial infarction [136], interacting with Annexin A2 to regulate smooth muscle cell activity and maintain vascular homeostasis in varicose veins [139], and binding to Smad3 to act as a molecular brake on TGF-β signaling in smooth muscle cell differentiation [140]. Furthermore, GAS5’s role in inflammatory conditions is quite complex, involving a direct interaction with either NF-κB or glucocorticoid receptors [12,141], with particular relevance in instances of steroid resistance. Lastly, GAS5 displays unique RNA–protein interactions in viral pathogenesis, such as acting as a decoy for viral NS3 [21] or regulating YBX1 in HBV-associated cancers [145]. These diverse functions position GAS5 as a crucial molecular regulator and a promising target for the development of novel therapeutic strategies against cancer, cardiovascular diseases, inflammatory disorders, and viral infections.

## 7. Conclusions

Through the evaluation of an extensive amount of research in this review, it is evident that GAS5 displays complex and context-dependent methods for regulating miRNAs and their downstream pathways across various pathological conditions. The ability of GAS5 to function as a ceRNA for multiple miRNAs positions it as a critical regulatory molecule with diverse implications for disease progression and potential therapeutic interventions.

In cancer, GAS5 consistently demonstrates its ability to function as a powerful tumor suppressor through many miRNAs, most notably through the well-characterized GAS5/miR-21 axis [13,31,32,35,37,38,39,40]. By modulating multiple classical oncogenic pathways such as PTEN/PI3K/AKT and SPRY/RAF/ERK, it regulates critical processes central to cancer progression such as EMT [42,43,56,57], chemoresistance [46,59,77], and metastasis [63,71]. This persistent anti-tumor activity of GAS5 across various cancer types suggests that strategies aimed at upregulating GAS5 expression could represent a promising therapeutic approach in oncology.

The role of GAS5 in cardiovascular diseases reveals a more nuanced relationship. While GAS5 can exacerbate conditions such as myocardial infarction [121] and atherosclerosis [103] through the promotion of apoptosis and inflammation, it also yields protective effects in specific contexts, such as mitigating cardiac fibrosis through the miR-217/SIRT1 pathway [112] and preventing EPC senescence via miR-223 regulation [114]. This duality implies the need for a careful consideration of context when developing GAS5-targeted therapies for cardiovascular conditions.

In inflammatory-associated diseases, the effects of GAS5 can typically be defined as having a context-dependent relationship, as described for cardiovascular diseases. Consistently, GAS5 shows detrimental effects in neurological conditions like cerebral ischemic stroke [82,88,99,104], Parkinson’s disease [108,115], and Alzheimer’s disease [98], where its inhibition could offer an improvement in patient outcomes; however, GAS5 also demonstrates protective effects in conditions such as NAFLD [100], rheumatoid arthritis [118], and diabetic nephropathy [113,119], where the suppression of GAS5 could have potentially detrimental effects. In fact, the case of sepsis particularly demonstrates this complexity, with GAS5 potentially playing different roles in inflammation modulation depending on the disease stage and cellular context [96,97,109,123], making GAS5 manipulation in this case particularly complicated. Despite this complexity, with further exploration, GAS5’s therapeutic potential could be elucidated.

In viral infections, GAS5 generally seems to play a protective role, which is particularly evident in HIV infection, where it can suppress viral replication through the sponging of miR-873 [122] and aid in restoring T cell function via miR-21 modulation [94]. Its potential as a biomarker of COVID-19 severity [111] and ability to suppress HCV replication [21] further underscore its significance in viral pathogenesis.

All things considered, the literature highlights GAS5’s conserved role as a ceRNA across varying pathological conditions, with its specific miRNA interactions and therapeutic implications varying by situation. While GAS5 upregulation demonstrates clear therapeutic potential in cancer, its effects in cardiovascular and inflammatory diseases rely heavily on the target cell type and context of the disease, allowing it to have either detrimental or protective functions. Future research should prioritize elucidating GAS5’s context-dependent roles, developing tissue-specific therapies, and exploring its potential as a biomarker. Targeting GAS5/miRNA axes holds significant promise for innovative treatments, but substantial success will require precise tailoring to best serve the disease context.

## Figures and Tables

**Table 1:** Summary of GAS5/miRNA-regulated pathways in human diseases.

miRNA	Targeted Pathway	Cell, Tissue, or Disease	Reference
miR-9	-FOXO3	-Cerebral ischemic stroke	[82]
miR-10a	-BDNF	-Asthma	[83]
miR-18a	-Undefined -CTGF	-Prostate cancer cells -Glioma cells -Mesenchymal stem cells	[51] [50] [84]
miR-21	-PDCD4/p21 or PTEN/PI3K/AKT -TLR4/NLRP3 -SYRP1/SPRY2/RAF/ERK -FGF1 -SMAD/BAX -TSP-1 -Undefined	-Acute myocardial infarction -Papillary thyroid carcinoma -Cervical cancer -Hepatocellular carcinoma -Breast cancer -Myocardial infarction-induced cardiomyocytes -Bladder cancer -Oral squamous cell carcinoma -Cardiac fibrosis -Non-small cell lung cancer -Ischemic brain injury -Laryngeal squamous cell carcinoma -Cardiomyocyte apoptosis -Glomerulosclerosis -Endometrial cancer -Glioblastoma -Vascular smooth muscle cells -Myocardial injury -Ovarian cancer -Temporomandibular joint osteoarthritis -Growth plate chondrocytes -Laryngeal squamous cell carcinoma -Lung cancer -Acute kidney injury -Osteosarcoma -Uveal melanoma -CD4 T cell senescence	[85] [62] [39] [32] [13] [86] [47] [45] [87] [46] [88] [48] [89] [35] [44] [38] [90] [91] [49] [37] [92] [48] [40] [93] [42] [43] [94]
miR-23a miR-23a-3p	-WT1 -ATG3 -PTEN/PI3K/AKT -TP63 -Undefined -E-cadherin -TLR4 -MiT-TFE/Nrf2 -PTEN/PI3K	-Ovarian cancer -HEK 293T cells -Hepatic fibrosis -Osteosarcoma cells -Non-small cell lung cancer -Non-small cell lung cancer -Glioblastoma -Sepsis -Sepsis -Alzheimer’s	[68] [66] [95] [61] [58] [67] [69] [96] [97] [98]
miR-26b-5p	-SMAD1	-Cerebral ischemia/reperfusion	[99]
miR-28a-5p	-MARCH7/NLRP3	-Nonalcoholic fatty liver disease	[100]
miR-32-5p	-PTEN	-Pancreatic cancer	[71]
miR-34a	-Undefined	-Common solid tumors	[81]
miR-103	-PTEN/AKT/mTOR	-Endometrial cancer	[70]
miR-106b	-IER3	-Cervical cancer	[75]
miR-135b miR-135a-5p	-Undefined -RECK -FOXO1 -KCNQ3 -Undefined	-Non-small cell lung cancer -Hepatocellular carcinoma -Osteoporosis -Epilepsy -Atherosclerosis	[80] [73] [101] [102] [103]
miR-137	-Notch-1	-Ischemic stroke	[104]
miR-142-5p	-TP53INP1 -SMAD3	-Cardiomyocytes under hypoxic conditions -Renal fibrosis	[105] [106]
miR-145	-Undefined	-Prostate cancer	[79]
miR-146a	-SMAD4	-Osteoarthritis	[107]
miR-150	-Fosl1/PTEN/AKT/mTOR	-Parkinson’s disease	[108]
miR-155-5p	SIRT1/HMGB1	-Sepsis	[109]
miR-181c-5p	-Hippo	-Pancreatic cancer	[77]
miR-182-5p	-FOXO3a	-Colorectal cancer cells	[76]
miR-194-3p	-TXNIP	-Coronary atherosclerosis	[110]
miR-196a-5p miR-196a	-FOXO1 -HOXA5 -Undefined	-Glioma stem cells -Cervical cancer -Ovarian cancer -Esophageal squamous cell carcinoma	[52] [54] [55] [53]
miR-200	-ACE-2	-COVID-19 severity	[111]
miR-205	-FOXO1	-Cervical cancer	[54]
miR-217	-SIRT1	-Cardiac fibrosis	[112]
miR-221	-SOCS3 -ARHI -SIRT1 -PUMA -DKK2/WNT -TP63 -SIRT1 -Undefined	-Pancreatic cancer -Osteosarcoma -Fibrosis in diabetic nephropathy -Neurons under hypoxic conditions -Breast cancer -Non-small cell lung cancer -Diabetic nephropathy -Atherosclerosis	[56] [57] [113] [27] [59] [58] [113] [26]
miR-222	-PTEN/PIK3/AKT -BMF/Bax	-Gastric cancer -B lymphocytic leukemia -Glioma cells	[64] [63] [60]
miR-223 miR-223-3p	-hZIP1 -NAMPT -NLRP3	-Renal cell carcinoma -Endothelial progenitor cells -Parkinson’s disease	[72] [114] [115]
miR-325	-CCT8	-Neonatal hydrocephalus	[116]
miR-335	-ROCK1/AKT/GSK-3β	-Myocardial ischemia/reperfusion injury	[117]
miR-361	-PDK4	-Rheumatoid arthritis	[118]
miR-424	-PRC2	-Malignant glioma	[78]
miR-452-5p	-NLRP3/Caspase-1	-Renal tubular cells	[119]
miR-455-5p	-SOCS3/JAK2/STAT3	-Macrophage polarization in childhood pneumonia	[120]
miR-525-5p	-CALM2	-Myocardial infarction	[121]
miR-873	-Undefined	-HIV-1 replication	[122]
miR-1323	-TP53INP1	-Hepatocellular carcinoma	[74]

## Abbreviations

miRNAs	MicroRNAs
LncRNAs	Long noncoding RNA
GAS5	Growth arrest-specific 5
PTEN	Phosphatase and tensin homolog
PIK3	Phosphoinositide 3-kinases
AKT	Protein kinase B
mTOR	Mammalian target of rapamycin
BAD	BCL2 associated agonist of cell death
p21	Cyclin-dependent kinase inhibitor 1
SPRY1	Sprouty RTK signaling antagonist 1
RAF	Rapidly accelerated fibrosarcoma
ERK	Extracellular-regulated kinase
PDCD4	Programmed cell death 4
SMAD	Sma- and Mad- related protein
SMAD7	SMAD family member 7
BAX	BCL2 associated X
BIM	Bcl-2-like protein 11
FGSC	Female germline stem cell
ATG3	Autophagy related 3
ATG8	Autophagy related 8
p62	Ubiquitin-binding protein
FOXO1	Forkhead box protein O1
MCF7	Michigan cancer foundation-7
HOXA5	Homeobox 5
GR	Glucocorticoid receptor
GSC	Glioma stem cell
PID1	Phosphotyrosine interaction domain containing 1
TP63	Tumor protein p63
SOCS3	Suppressor of cytokine signaling 3
PUMA	P53-upregulated modulator of apoptosis
JNK	c-Jun N-terminal kinase
H2AX	H2A histone family member X
SIRT1	Sirtuin 1
ARHI	Aplasia Ras homolog member I
ABCB1	P-glycoprotein
DKK2	Dickkopf 2
BMF	BCL-2-modifying factor
NAMPT	Nicotinamide phosphoribosyltransferase
JAK2	Janus kinase 2/signal transducer
STAT3	Activator of transcription 3
TP53INP1	Tumor protein p53 inducible nuclear protein 1
CDK4	Cyclin-dependent kinase 4
BCL-2	B-cell lymphoma 2
ceRNA	Competing endogenous RNA
ROCK1	Rho-associated protein kinase 1
YAP1	Yes-associated protein
GSK-3β	Glycogen synthase kinase 3
mPTP	Mitochondrial permeability transition pore
UM	Uveal melanoma
CALM2	Calmodulin 2
CIS	Cerebral ischemic stroke
FOXO3	Forkhead box protein O3
SMAD1	SMAD family member 1
MCAO	Middle cerebral artery occlusion
OGD	Oxygen-glucose deprivation
NLRP3	NOD-like receptor family pyrin domain containing 3
Caspase-1	Cysteinyl aspartate-specific protease
IL-1β	Interleukin-1β
IL-18	Interleukin-18
KCNQ3	Potassium Voltage-Gated Channel Subfamily Q Member 3
ox-LDL	Oxidized low-density lipoprotein
apoE	Apolipoprotein E
HFD	High-fat diet
AS	Atherosclerosis
BMSCs	Bone marrow mesenchymal stem cells
BDNF	Brain-derived neurotrophic factor
TSP-1	Thrombospondin-1
EMT	Epithelial to mesenchymal transition
H/R	Hypoxia/reoxygenation
TXNIP	Thioredoxin-interacting protein
EC	Endothelial cell
EPC	Endothelial progenitor cell
TLR4	Toll-like receptor 4
Notch-1	Neurogenic locus notch homolog protein 1
PD	Parkinson’s Disease
ASC	Apoptosis-associated speck-like protein containing a CARD
Aβ	Amyloid beta
CCT8	Chaperonin containing TCP1 subunit 8
TGF-β	Transforming growth factor β
LPS	Lipopolysaccharide
MiT/TFE	Microphthalmia/transcription factor E
MARCH7	Membrane-associated ring-CH finger protein 7
HMGB1	High mobility group box 1
NRF2	Nuclear factor erythroid 2–related factor 2
NAFLD	Nonalcoholic fatty liver disease
RA	Rheumatoid arthritis
PDK4	Pyruvate dehydrogenase kinase 4
OA	Osteoarthritis
DN	Diabetic nephropathy
HIV	Human immunodeficiency virus
Gag	Group-specific antigen
HCV	Hepatitis C virus
Fosl1	FOS like antigen 1
E2F1	E2F transcription factor 1
APC	Adenomatous polyposis coli
EVs	Extracellular vesicles
CDKN1C	Cyclin dependent kinase inhibitor 1C
GRE	Glucocorticoid response element
Sema3a	Semaphorin 3a
GSVs	Great saphenous veins
YBX1	Y-box-binding protein 1
rSBEs	RNA Smad-binding elements
RIP	RNA immunoprecipitation
SMC	Smooth muscle cell
PBMCs	Peripheral blood mononuclear cells
INS	Idiopathic nephrotic syndrome
SRNS	Steroid-resistant nephrotic syndrome
